# Atrial Fibrillation, Cognitive Decline, and Dementia: an Epidemiologic Review

**DOI:** 10.1007/s40471-018-0159-7

**Published:** 2018-07-07

**Authors:** Mozhu Ding, Chengxuan Qiu

**Affiliations:** 1 Aging Research Center, Department of Neurobiology, Care Sciences and Society, Widerströmska Huset, Karolinska Institutet and Stockholm University, Tomtebodavägen 18A, 171 65 Solna, Sweden; 2 Department of Neurology, Shandong Provincial Hospital Affiliated with Shandong University, Jinan, Shandong China

**Keywords:** Atrial fibrillation, Cognitive decline, Dementia, Pharmaceutical treatment, Epidemiology

## Abstract

**Purpose of Review:**

Atrial fibrillation (AF) and dementia are both prevalent diseases in aging societies, which exert a great economic burden worldwide. Although a handful of epidemiologic studies have indicated that AF is independently associated with faster cognitive decline and a higher risk of dementia, there is still a lack of comprehensive understanding of the observed association. In this review, we summarize evidence from major epidemiologic studies concerning AF-related cognitive decline and dementia, the potential mechanisms underlying their association, and the cognitive benefits of treatment options.

**Recent Findings:**

A large majority of population-based longitudinal studies have consistently shown an independent association of AF with cognitive decline and dementia with varying effect sizes, depending on the age of the study population and the presence of clinical stroke. The underlying pathways linking AF to cognitive phenotypes may involve systemic inflammation, cerebral hypoperfusion, and cerebral small vessel disease and microemboli. However, current evidence is insufficient to support the potential benefits of AF treatment in reducing risk of cognitive decline and dementia.

**Summary:**

Current epidemiologic research suggests that AF contributes to cognitive decline and dementia, independent of a history of stroke. Further work is warranted to elucidate the potential mechanisms underlying this association, and more well-designed studies are needed to explore the possible cognitive benefits of different therapeutic options in patients with AF.

## Introduction

Atrial fibrillation (AF) is the most common sustained cardiac arrhythmia, characterized by uncoordinated atrial activation and ineffective atrial contraction. AF can be defined on electrocardiogram (ECG) as irregular RR intervals and absence of distinct P waves [1]. With a lifetime risk of around 25% in people aged 55 years and above [2, 3], AF predominantly affects older adults and represents a major clinical risk factor for heart failure, myocardial infarction, ischemic stroke, and mortality [4–6]. Since the 1990s, the prevalence, incidence, and overall burden of AF have been continuously growing worldwide [7]; it was estimated that approximately 46.3 million people worldwide had AF in 2016, a 40% increase from 33.5 million in 2010 [7, 8].

Dementia is a disabling syndrome of the brain, characterized by a progressive deterioration of memory and other cognitive functions [9]. The number of people affected by dementia worldwide was estimated at 47 million in 2015, and each year around 7.7 million new cases were added into the pool [10]. The total global cost of dementia was estimated at US$818 billion in 2015 and will rise above US$1 trillion in 2018 [10]. Thus, dementia has been recognized by WHO, Alzheimer’s Disease International, and the London G8 Dementia Summit as a global public health priority [11, 12]. In the past decade, AF has emerged as a potential risk factor for cognitive decline and dementia; however, there is still a lack of a conclusive understanding of the complex relationship between these two disorders. Knowledge gaps exist regarding the mechanisms of AF-related cognitive consequences beyond aging and stroke, and the optimal way to prevent cognitive decline and dementia in patients with AF remains to be established [13••]. Understanding the pathophysiological mechanisms behind this association and exploring the cognitive benefits of different treatment options in patients with AF may help reduce the massive burden of both AF and dementia on individuals and health care systems.

Therefore, in this review, we aim to summarize the current epidemiologic literature on (1) the association of AF with cognitive decline and dementia, (2) plausible mechanisms underlying this association, and (3) the potential cognitive benefit of therapeutic options in patients with AF.

## Association of Atrial Fibrillation with Cognitive Decline and Dementia: Epidemiologic Evidence

A number of well-established population-based cohort studies have demonstrated an association between AF and increased risk of cognitive decline or dementia (Table 1) [14, 15••, 16••, 17••, 18, 19, 20••, 21••, 22–27], especially among younger-old people. For instance, the UK Whitehall II study showed that in people aged 45–69 years, AF is significantly associated with higher risk of incident dementia (HR = 1.87, 95% CI 1.37–2.55) and that longer exposure to AF is associated with faster cognitive decline compared with AF-free adults [16••]; taking into account incident stroke did not significantly alter the results. In the Atherosclerosis Risk in Communities (ARIC) Study, data were available from 12,515 participants (mean age 56.9 years) who were followed up for over 20 years. This study reported that incident AF was associated with faster global cognitive decline and higher risk of dementia after adjusting for cardiovascular diseases including ischemic stroke [15••]. The US Cardiovascular Health Study demonstrated that, in the absence of clinical stroke, the cognitive function declined faster in patients experiencing incident AF compared to people with no prior AF [19]. In the Rotterdam study, AF is associated with an elevated risk of incident dementia only among people younger than 67 years (HR = 1.81, 95% CI 1.11–2.94) [17••]. Similarly, the US Intermountain Heart Collaborative Study showed that the highest risk of dementia associated with AF was seen in people younger than 70 years [24]. Concerning dementia subtypes, in a large-scale population-based study, we found that AF is associated with increased risk of vascular and mixed dementia but not with Alzheimer’s disease (AD). Among the oldest-old (e.g., age ≥ 80 years), there is yet not enough evidence from epidemiologic studies to support an association of AF with cognitive decline and dementia, and more data from population-based studies are needed to fully elucidate this association in the oldest-old population, as reported by a recent review of studies carried out in subjects ≥ 80 years [28•].

**Table 1:** A summary of the main population-based longitudinal studies assessing the association between atrial fibrillation and risk of cognitive decline or dementia

Study, country	Study population	Assessment of atrial fibrillation	Assessment of cognitive outcomes	Main findings
Nishtala et al. [14], USA	Framingham Heart Study original and offspring cohort; 3- or 6-year follow-up; mean age 83 for the original and 68 for the offspring cohort; *N* = 2628	Self-reports, ECG, and medical registers	Cognitive function: a neuropsychological battery on major cognitive domains	• Prevalent AF is associated with faster decline in executive function (*β* coefficient = −0.31; 95% CI −0.37, −0.25)
Chen et al. [15••], USA	Atherosclerosis Risk in Communities Study; 20-year follow-up; mean age 56.9 at baseline; *N* = 12,515	ECG and ICD-9 codes (427.31 and 427.32)	Cognitive function: 3 neuropsychological tests Dementia: diagnostic algorithm and ICD-9 codes	• AF is associated with faster global cognitive decline (difference in *Z* score = 0.12, 95% CI 0.03–0.23) • AF is associated with incident dementia (HR = 1.31, 95% CI = 1.11–1.55)
Singh-Manoux et al. [16••], UK	Whitehall II Study; 26.6-year follow-up; age range 45–69 at baseline; *N* = 10,217	12-lead ECG and ICD-9/10 codes (437.3 and I48)	Cognitive function: a cognitive test battery (memory, reasoning, and verbal fluency) Dementia: ICD-10 codes	• Longer exposure to AF is associated with faster cognitive decline (*p* for trend = 0.01) • AF is associated with incident dementia (HR = 1.87, 95% CI 1.37–2.55) • Stroke does not explain these associations
de Bruijn et al. [17••], The Netherlands	Rotterdam Study; 21-year follow-up; age 55+ at baseline; *N* = 6514	ECG, physician diagnosis, and medical registers	Dementia: DSM-III-R criteria	• Incident AF is related to dementia only in people < 67 years (HR = 1.81, 95% CI 1.11–2.94) • Duration of AF is associated with dementia risk only in people < 67 years (*p* for trend = 0.003)
Rusanen et al. [18], Finland	CAIDE study; mean 7.8-year follow-up; age range 65–79; *N* = 1510	Medical registers	Dementia: DSM-IV criteria	• Prevalent AF is associated with dementia (HR = 2.61, 95% CI 1.05–6.47) and Alzheimer’s disease (HR = 2.54, 95% CI 1.04–6.16); the association is evident only in APOE non-carriers
Thacker et al. [19], USA	Community-dwelling people; mean 7-year follow-up; age 73 at baseline; *N* = 5150	ECG, ICD-9 codes	Cognitive function: modified MMSE (3MSE) and Digit Symbol Substitution Test	• Incident AF is associated with accelerated 5-year cognitive decline for age 70, 75, 80, and 85 years
Haring et al. [20], USA	RCTs of postmenopausal women; median 8.6-year follow-up; age 60+ at baseline; *N* = 7479	Self-reports or physical measure	MCI and probable dementia: DSM-IV criteria	• Prevalent AF is not related to probable dementia (HR = 1.12, 95% CI 0.59–2.14) • Prevalent AF is not associated with mild cognitive impairment (HR = 1.46, 95% CI 0.90–2.37)
Marzona et al. [21], 40 countries	Two RCTs of patients with CVD or diabetes; median follow-up 56 months; mean age 66.5 at baseline; *N* = 31,506	12-lead ECG	Cognitive function: MMSE Dementia: new dementia diagnosis, reported severe cognitive impairment, or MMSE ≤ 23	• Prevalent and incident AF is associated with ≥ 3 points decline in MMSE during the follow-up (HR = 1.14, 95% CI 1.03–1.26) • Prevalent and incident AF is associated with dementia (HR = 1.30, 95% CI 1.14–1.49)
Dublin et al. [22], USA	Community-dwelling people; mean 6.8-year follow-up; mean age 74.3 at baseline; *N* = 3045	At least two documented ICD-9 codes within 12 months	Dementia: DSM-IV criteria AD: NINCDS-ADRDA criteria	• Prevalent AF is associated with dementia (HR = 1.38, 95% CI 1.10–1.73) • Prevalent AF is associated with AD (HR = 1.50, 95% CI 1.16–1.94)
Marengoni et al. [23], Sweden	Kungsholmen Project; 6-year follow-up; age 75+ at baseline; *N* = 685	Physician diagnosis, medical records, drug use, and ICD-9 codes	Dementia: DSM-III-R criteria	• No association between AF and dementia (HR = 0.9, 95% CI 0.5–1.7) or AD (HR = 0.8, 95% CI 0.4–1.5)
Bunch et al. [24], USA	Health care patients; mean 5-year follow-up; mean age 60.6 at baseline; *N* = 37,025	ECG and ICD-9 codes	Dementia: ICD-9 codes	• Prevalent AF is associated with vascular dementia (HR = 1.73, *p* = 0.001), senile dementia (HR = 1.39, *p* = 0.005), and non-specific dementia (HR = 1.44, *p* < 0.001); highest risk was in younger group (< 70 years)
Peters et al. [25], UK	RCT of hypertensive patients; mean 2-year follow-up; age 80+ at baseline; *N* = 3336	ECG	Cognitive decline: decrease to MMSE < 24 or by > 3 point annually Dementia: DSM-IV criteria, a CT scan, and modified ischemic score	• No association between prevalent AF and dementia (HR = 1.03, 95% CI 0.62–1.72) • No association between prevalent AF and cognitive decline (HR = 1.08, 95% CI 0.80–1.46) • No association between prevalent AF and annual change of MMSE (*β* coefficient = −0.26; 95% CI −0.66, 0.13)
Rastas et al. [26], Finland	Community-dwelling people; 9-year follow-up; age 85+ at baseline; *N* = 553	12-lead ECG or 1-h Holter ECG; health records	Dementia: DSM-III-R criteria	• No association between prevalent AF and dementia
Tilvis et al. [27], Finland	Community-dwelling people; 10-year follow-up; age 75, 80, and 85 at baseline; *N* = 650	Clinical examinations	Cognitive decline: increase in Clinical Dementia Rating class or at least 4 point decrease in MMSE	• AF is associated with 5-year cognitive decline (RR = 2.88, 95% CI 1.26–6.06)

*AF* atrial fibrillation, *ECG* electrocardiogram, *HR* hazard ratio, *CI* confidence interval, *RR* relative risk, *OR* odds ratio,* ICD-9/10* International Statistical Classification of Diseases and Related Health Problems, 9th Revision/10th revision, *DSM-III-R* Diagnostic and Statistical Manual of Mental Disorders, 3rd Edition, Revised, *DSM-IV* Diagnostic and Statistical Manual of Mental Disorders, 4th Edition, *NINCDS-ADRDA* National Institute of Neurological and Communicative Disorders and Stroke-Alzheimer's Disease and Related Disorders Association, *MCI* mild cognitive impairment, *CAIDE* Cardiovascular Risk Factors, Aging and Dementia. *MMSE* Mini-Mental State Examination, *RCT* randomized control trial, *CVD* cardiovascular disease, *CT* computed tomography

Several systematic reviews and meta-analyses have been conducted to assess the relationship between AF and dementia. For example, a systematic review comprising data from 15 longitudinal population-based studies that cover 46,637 participants reported that the association between AF and incident dementia is evident mainly in studies solely focusing on patients with stroke (HR = 2.4, 95% CI 1.7–3.5), while their association remained marginal in studies with broader populations (HR = 1.6, 95% CI 1.0–2.7) [29]. Another meta-analysis including 8 longitudinal studies with 77,668 patients not suffering from acute stroke at baseline indicated that AF was independently associated with an increased risk of dementia (HR = 1.4, 95% CI 1.2–1.7) [30]. A larger systematic review including 21 studies with either cross-sectional or longitudinal designs demonstrated that AF is associated with more than a twofold increased risk of dementia or cognitive impairment after stroke (HR = 2.7, 95% CI 1.8–4.0), and the increased risk, although less strong, remained significant when restricting to studies that only included participants without a history of stroke (HR = 1.4, 95% CI 1.1–1.7) [31].

Taken together, current evidence from population-based longitudinal studies and systematic reviews suggests an independent association of AF with cognitive decline and dementia, especially among young-old people, irrespective of a history of stroke. Further understanding the potential pathological mechanisms behind this association may help develop proper preventive and therapeutic interventions to counteract the risk of cognitive consequences resulting from AF.

## Association of Atrial Fibrillation with Cognitive Decline and Dementia: Potential Mechanisms

**Fig. 1:**
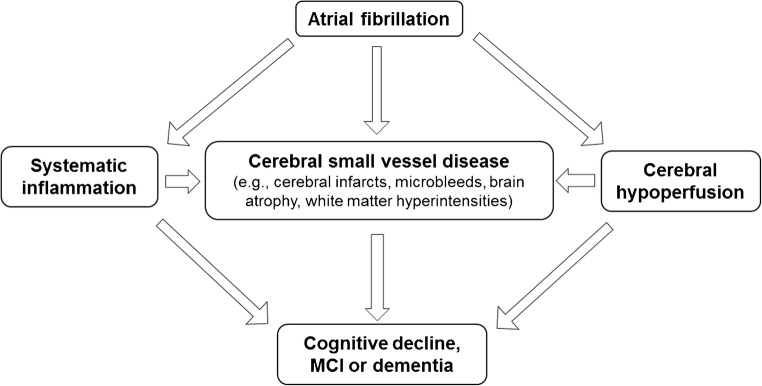
Potential pathophysiological pathways linking atrial fibrillation with cognitive decline, mild cognitive impairment (MCI), and dementia

It has been argued that AF and dementia may merely develop alongside each other as a result of the aging process and shared precipitating conditions. Indeed, AF, cognitive decline, and dementia share several modifiable risk factors, such as lifestyle factors (e.g., physical inactivity, smoking, and excessive alcohol consumption), cardiometabolic risk factors (e.g., obesity, hypertension, diabetes, and high cholesterol), and cardiovascular disorders (e.g., atherosclerosis, heart failure, and coronary heart disease) [9, 32, 33]. Although major clinical disorders have been adjusted for in the majority of studies investigating the association between AF and cognitive decline or dementia, imperfect measurements of these disorders and other unknown risk factors may still confound the association. Beyond shared risk factors, AF may accelerate cognitive decline and increase the risk of dementia through different pathways such as cerebral hypoperfusion, systemic inflammation, and cerebral small vessel diseases (SVDs) (Fig. 1) [32, 34–36].

### Cerebral Hypoperfusion

Persistent AF reduces cardiac output and may lead to chronic cerebral hypoperfusion and hypoxia, which may impair the clearance and promote the accumulation of amyloid-beta peptides in cerebral vessels, thus leading to cerebral amyloid angiopathy and contributing to the onset of Alzheimer’s disease (AD) [37–39]. However, this hypothesis has been investigated by very few studies, among which one study examined the cognitive function and cerebral blood flow velocity in the major brain arteries in 187 patients with heart failure, out of which 32% had a history of AF [40]. This cross-sectional study showed a greater reduction in cerebral blood flow and worse cognitive performance among heart failure patients with AF than those without AF, as well as an association between decreased cerebral perfusion and worse cognitive function. However, it has also been argued in a recent review that the cerebral hypoperfusion hypothesis cannot explain the majority of dementia cases since cerebral autoregulation is expected to maintain cerebral blood flow within a wide blood pressure range and evidence is lacking to support that cerebral autoregulation is impaired in patients with AF [32]. Further studies are needed to investigate whether and how cerebral hypoperfusion plays a role in the association between AF and cognitive dysfunction.

### Systemic Inflammation

Several comprehensive reviews have summarized the potential pathways linking the initiation and perpetuation of AF to systemic inflammation status, including but not limited to increased level of C-reactive protein, tumor necrosis factor-α, interleukin (IL)-2, IL-6, and IL-8, and monocyte chemoattractant protein-1 [41, 42]. Previous studies have shown that these inflammation markers are associated with hypercoagulation, endothelial dysfunction, and increased platelet activation, thus contributing to AF-related thromboembolism [43, 44].

Given the high systemic inflammation status and prothrombotic environment in AF, it is possible that patients with AF are more susceptible to blood-brain barrier damage and cerebral microstructural changes, leading to cognitive decline and dementia [45]. It has been shown that a higher level of high-sensitivity C-reactive protein is associated with worse executive functioning and more microvascular damage in the white matter [46], as well as an elevated risk of all dementia subtypes independently of cardiovascular risk factors and related diseases [47]. So far, the mediating role of inflammation in the association of AF with cognitive decline and dementia remains to be investigated.

### Cerebral Small Vessel Diseases and Microemboli

Cerebral SVDs refer to a group of pathological processes that affect the small arteries, arterioles, venules, and capillaries of the brain [48, 49]. Cerebral SVDs, as detected using brain imaging techniques such as magnetic resonance imaging, are subcortical infarcts, lacunes, white matter hyperintensities (WMH), brain atrophy, cerebral microbleeds (CMBs), and perivascular spaces [50]. Importantly, recent epidemiologic studies have shown that cerebral SVDs are a leading cause of cognitive decline and functional loss in the elderly [48, 51], and have been hypothesized to play a key role in the association of AF with cognitive decline and dementia [35].

Although cerebral infarction manifested as a stroke is the most feared consequence of AF, subclinical silent cerebral infarcts (SCIs) are found in up to 90% of people with AF [35]. A recent meta-analysis showed that AF was associated with more than a twofold increased risk of any SCIs, independent of AF subtypes [52], and presence of SCIs is associated with two to threefold increased risk of both symptomatic stroke and dementia [53, 54]. Consequently, a higher proportion of SCIs in patients with AF may put them at greater risk of cognitive decline and dementia. The ARIC study, in which 935 stroke-free participants were recruited and followed up for 10 years, showed that incident AF was associated with greater decline in word fluency among people who had prevalent SCIs; among those without prevalent SCIs, incident AF was not associated with cognitive decline [55]. These findings support the hypothesis that, in the absence of overt stroke, the association between AF and cognitive decline may be, at least partly, explained by SCIs.

On the other hand, the association between AF and WMH is less clear. For instance, one retrospective cohort study of 234 patients with stroke showed that AF was associated with the presence of anterior subcortical WMH [56•]. Similarly, another hospital-based study demonstrated that deep and subcortical WMH grade was higher among patients with AF compared to matched controls [57]. On the contrary, the Framingham Offspring Study found no evidence of an association between AF and WMH volumes over 6 years of follow-up after adjusting for several vascular risk factors and cardiovascular diseases [58]. Indeed, it has been suggested that brain ischemia resulting from low cardiac output and cerebral hypoperfusion may lead to WMH and that chronic SCIs could also convert into WMH [59, 60]. Yet whether and to what extent WMH play a role in the association of AF with cognitive decline and dementia remains to be further investigated.

AF appears to be also associated with loss of brain volumes, which in turn leads to cognitive dysfunction. The underlying mechanisms may include hypoperfusion of gray matter, systemic inflammation, chronic micro-embolisms, and CMBs [61–63]. The cross-sectional study in Germany showed that AF is associated with reduced hippocampal volume, a hallmark in AD [64]. The population-based AGES-Reykjavik Study found that AF was associated with lower total brain, gray matter, and white matter volumes, especially among patients with longer duration of AF, but the association of AF with WMH volume was less evident [65]. The Framingham Offspring study demonstrated that AF was associated with lower frontal brain volume even after adjusting for vascular risk factors and APOE ε4 status [58].

As the brain ages, cerebral small vessels are susceptible to microbleeds partly due to cerebral amyloid angiopathy, especially when using anticoagulant treatment. The occurrence of CMBs increases with advancing age and is more common among AD and stroke patients [63]. A clinic-based study showed that CMBs were more common in patients with AF than those without [66]. Controversies exist regarding anticoagulation in older AF patients, since CMBs and subsequent intracranial hemorrhage are the most feared complications of anticoagulant treatment [67]. Interestingly, a clinical-based study of 550 patients with ischemic stroke and AF showed that a higher CHADS_2_ score (a score contains major clinical risk factors for stroke that is scored 1 point for each of chronic heart failure, hypertension, age ≥ 75 years, and diabetes and 2 points for ischemic stroke or transient ischemic attacks) was strongly correlated with the number of CMBs, and that the presence of CMBs was independently associated with the development of intracranial hemorrhage [68]. Furthermore, the Rotterdam study found that the presence of CMBs was associated with faster cognitive decline and higher risk of dementia and AD [69].

Taken together, current evidence suggests an important role of cerebral SVDs in the association between AF and cognitive dysfunction.

## Do Treatment Options in Patients with Atrial Fibrillation Prevent Cognitive Decline or Dementia?

### Anticoagulant Treatment

Oral anticoagulant drugs remain the first-line medication for the prevention of ischemic stroke in patients with AF [70]. Given that both macro-embolic (e.g., clinical stroke) and micro-embolic events (e.g., SCIs) related to AF may contribute to cognitive decline and the onset of dementia, it is likely that anticoagulant treatment could benefit cognitive function and lower the risk of dementia in AF patients by reducing cerebral infarct burden [71–73]. Warfarin is the most commonly prescribed anticoagulant agent for the prevention of AF-related stroke, even after the release of novel oral anticoagulant drugs (NOACs) [74]. Although a systematic review did not demonstrate an association between use of warfarin and risk of dementia in AF patients [75•], a few recent studies did show a trend toward a protective effect of warfarin therapy on cognitive outcomes in patients with AF [76••, 77•, 78•, 79^•^]. A retrospective study of all patients diagnosed with AF from 2006 to 2014 in the Swedish patient register reported that patients treated with anticoagulants had a 29% lower risk of dementia than those without anticoagulant treatment [76••]. Another community-based cohort study of patients with incident AF also demonstrated that warfarin therapy was associated with a 20% reduction in dementia risk over 5 years of follow-up [77•]. What confounds the interpretation of the effect of warfarin on dementia risk is that cognitive deficits may negatively influence the safety and thus the prescription decision of anticoagulant therapy in patients with AF. Furthermore, previous studies merely focus on the presence or absence of anticoagulant therapy and lack data on the quality of anticoagulation control, which is of major importance among older adults. It was reported that cognitive dysfunction was related to lower percentage time in therapeutic range (TTR) in older AF patients taking warfarin [80], and that decreasing percentage of TTR was associated with an increased risk of dementia [81]. It appears that both under-anticoagulation (i.e., international normalized ratio (INR) < 2) and over-anticoagulation (i.e., INR > 3) are associated with higher risk of dementia [81], probably due to cumulative brain injury from cerebral infarcts and microbleeds [34].

Although evidence is scarce regarding the effect of NOAC therapy on cognitive decline or dementia in patients with AF, it is plausible that use of NOACs could be associated with lower dementia risk compared to warfarin. In randomized-controlled clinical trials, NOACs have shown similar efficacy as warfarin with regard to prevention of stroke and are associated with lower rates of intracranial hemorrhage and death in patients with AF [82, 83]. Two retrospective register-based studies have recently indicated that use of NOACs was associated with a lower risk of dementia compared to warfarin in patients with AF [76••, 78•]. Large longitudinal studies with longer follow-up time are needed to clarify the effect of NOACs on cognitive function, and currently several randomized-controlled clinical trials focusing on cognitive outcomes in patients with AF have been initiated [32].

### Other Therapies

Low-dose antiplatelet drugs, such as aspirin, are often prescribed for the prevention of cardiovascular events in people at cardiovascular risk because of its antithrombotic properties [84]. However, evidence of effective prevention of stroke with antiplatelet drugs is weak in AF patients, with a potential for harm (e.g., increased risk of major bleeding or intracranial hemorrhage) [85]. Therefore, current international consensus guidelines recommended that use of antiplatelet drugs in AF patients should be limited, especially in older patients with AF [70]. In addition, previous studies have failed to show a protective effect of antiplatelet drugs on cognitive function in old age. For instance, a recent systematic review and meta-analysis that included 8 observational and interventional studies and 36,196 cognitively intact participants at baseline showed that chronic use of low-dose aspirin was not associated with global cognitive function, cognitive impairment, or the onset of dementia [86].

Since systemic inflammation has been proposed to be one of the potential mechanisms underlying the link between AF and cognitive dysfunction, it is likely that anti-inflammatory therapy may preserve cognitive function in patients with AF. Statins are now widely accepted to have anti-inflammatory properties, in addition to a lipid-lowering effect [87]. One register-based study in Taiwan reported a 20% decreased risk of incident non-vascular dementia in 51,253 patients with AF who received statin treatment compared to those who were not prescribed statin therapy [88•]. Another study showed that among older patients with AF, intensive lipid-lowering therapy with atorvastatin and ezetimibe was associated with less amygdala and hippocampal atrophy compared to placebo [89]. Nevertheless, these results need to be further confirmed in larger longitudinal studies.

With regard to rate and rhythm-control strategies, analysis of a subsample of the Atrial fibrillation Follow-up Investigation of Rhythm Management (AFFIRM) study showed no differences in global cognitive function between the rate-control and rhythm-control group at 2-month and yearly follow-up visits [90]. The Intermountain Atrial Fibrillation Study including 4212 patients who underwent catheter ablation for AF showed that 0.2% of the AF ablation patients developed AD after 3 years of follow-up compared to 0.9% in unoperated AF patients and 0.5% in patients without AF (*p* < 0.001) [91]. Similarly, differences were also observed for other dementia subtypes. These findings suggest that rhythm-control strategy might potentially suppress hypoperfusion-induced AD pathology [39].

Taken together, chronic use of low-dose antiplatelet drugs does not demonstrate a protective effect on cognitive function and is not recommended in patients with AF for stroke prevention for maintaining cognitive function unless anticoagulant drugs are contraindicated. Evidence is still sparse regarding whether anti-inflammatory treatment and rhythm- and rate-control therapy can truly preserve cognitive function and reduce the risk of dementia in patients with AF, and large longitudinal studies in more controlled settings are needed. In addition to pharmaceutical treatment, a recent expert consensus has recommended that general health maintenance and appropriate management of multiple risk factors and concomitant conditions related to AF (e.g., heart failure, hypertension, obesity, diabetes, and sleep apnoea) may decrease the risk of both AF and stroke, with a putative beneficial effect on cognitive function [13••].

## Conclusion and Future Directions

AF and dementia both affect predominantly the older population and exert a great economic and societal burden on aging societies. Numerous epidemiologic studies have shown an association of AF with an increased risk of cognitive decline and dementia with varying effect sizes, depending on the age of the study population and the presence of clinical stroke. Cerebral hypoperfusion, systemic inflammation, and cerebral SVDs are the major potential mechanisms underlying this association, and it is likely that these pathways work together to confer an increased risk of dementia in patients with AF. Efforts have been made to investigate whether different treatment strategies may counteract the risk of cognitive decline and dementia owing to AF; however, evidence is still sparse in this area. High-quality population-based longitudinal studies and randomized-controlled clinical trials are needed to explore different treatment options to prevent or delay cognitive decline and the onset of dementia in patients with AF.
